# Amorphous martensite in β-Ti alloys

**DOI:** 10.1038/s41467-018-02961-2

**Published:** 2018-02-06

**Authors:** Long Zhang, Haifeng Zhang, Xiaobing Ren, Jürgen Eckert, Yandong Wang, Zhengwang Zhu, Thomas Gemming, Simon Pauly

**Affiliations:** 1 Shenyang National Laboratory for Materials Science, Institute of Metal Research, Chinese Academy of Sciences, 72 Wenhua Road, Shenyang, 110016 China; 2 IFW Dresden, Institute for Complex Materials, Helmholtzstraße 20, 01069 Dresden, Germany; 3 Multi-disciplinary Materials Research Centre, Frontier Institute of Science and Technology, Xi’an Jiaotong University, Xi’an, 710049 China; 4 Ferroic Physics Group, National Institute for Materials Science, Tsukuba, 305-0047 Japan; 5 Erich Schmid Institute of Materials Science, Austrian Academy of Sciences, Jahnstraße 12, 8700 Leoben, Austria; 6 Department Materials Physics, Montanuniversität Leoben, Jahnstraße 12, 8700 Leoben, Austria; 7 State Key Laboratory for Advanced Metals and Materials, University of Science and Technology Beijing, Beijing, 100083 China

## Abstract

Martensitic transformations originate from a rigidity instability, which causes a crystal to change its lattice in a displacive manner. Here, we report that the martensitic transformation on cooling in Ti–Zr–Cu–Fe alloys yields an amorphous phase instead. Metastable β-Ti partially transforms into an intragranular amorphous phase due to local lattice shear and distortion. The lenticular amorphous plates, which very much resemble α′/α″ martensite in conventional Ti alloys, have a well-defined orientation relationship with the surrounding β-Ti crystal. The present solid-state amorphization process is reversible, largely cooling rate independent and constitutes a rare case of congruent inverse melting. The observed combination of elastic softening and local lattice shear, thus, is the unifying mechanism underlying both martensitic transformations and catastrophic (inverse) melting. Not only do we reveal an alternative mechanism for solid-state amorphization but also establish an explicit experimental link between martensitic transformations and catastrophic melting.

## Abstract (web summary)

Displacive martensitic transformations through lattice distortion usually involve a change from one crystal structure to another. Here however, the authors “melt” metastable Ti alloys during cooling and show that a martensitic transformation can lead to the formation of an intragranular amorphous phase.

## Introduction

Amorphous materials are generally obtained by quenching liquids in order to avoid crystallization^1,2^ and multicomponent or even monatomic metallic glasses can be prepared in this way^3^. Alternatively, amorphous materials can also be produced through a transformation from crystalline solids, known as solid-state amorphization (SSA)^4^. A variety of processes can fully or partially transform crystals into an amorphous phase, among them high-pressure treatments^5–8^, irradiation with ions or electrons^9^, severe plastic deformation^10^, inter-diffusion in multilayers^11,12^, mechanical alloying^13^, hydrogen absorption^14^, or decompression of solids^15–17^. It has even been reported that a metastable liquid is the intermediate state in certain solid–solid phase transformations^18,19^.

From a thermodynamic viewpoint, SSA can be considered a melting process far below the equilibrium melting temperature^20,21^. One requirement for solid-state amorphization^4^ is the generation of a metastable crystalline state containing a high density of lattice defects^11,22,23^. The continuous increase of lattice defects augments the static disorder up to a critical point at which the lattice becomes unstable and a glass forms^9,23^. Analogies between SSA and melting have been widely recognized^20,21,24^ and SSA without compositional change (polymorphic SSA) on cooling of a crystalline solid is known as inverse melting^17,24,25^. Such a polymorphic or (more appropriately) congruent inverse melting has been observed in various materials, such as polymers^26^, silicon^10,27^, and Ti–Cr supersaturated alloys^22^. Polymorphic SSA generally proceeds via nucleation and growth, i.e., the amorphous phase nucleates at lattice defects followed by its growth resulting from a gradual collapse of the crystal lattice^4,12,19^.

Similarly, the process of nucleation and growth also holds for conventional melting of crystals and the liquid begins to form at defects^19,28,29^, such as surfaces or internal lattice defects once the equilibrium melting temperature is reached. However, a different, rarely encountered mode of melting has been long been predicted to occur in defect-free crystals undergoing substantial superheating via a rigidity catastrophe^29–31^. Several upper limits for predicting the onset of catastrophic melting have been postulated in the past decades, including an isochoric limit (i.e., the superheated crystal and liquid have the equal volume)^31^, an isenthalpic limit (equal enthalpy)^32^ and an isentropic limit (equal entropy)^31,32^. Yet, since real solids generally cannot be superheated to temperatures at which catastrophic melting sets in, these limits for catastrophic melting have not been reached in experiments^33^. Consequently, it remains an open question, which limit provokes catastrophic melting^29,34^. In this context, congruent inverse melting can provide helpful insights because no superheating of the solid is required, which greatly facilitates experimental access.

Martensitic transformations are another very common phenomenon and occur in a variety of materials^35,36^, including steels^37^, polymers^38^, ceramics^39^, shape memory alloys^35^ as well as Ti, and Zr alloys^36^. During cooling or deformation, phonon anomalies accompanied by elastic softening and lattice shear cause a lattice instability, which transform the parent lattice in a diffusionless and displacive manner into a new crystal lattice^36,40^. Such an elastic softening and lattice shear have also been postulated based on theoretical consideration^30^ to account for polymorphic SSA and catastrophic melting, which has later been corroborated by molecular dynamic simulations^29,41,42^. However, a possible link between martensitic transformation and catastrophic (inverse) melting has escaped experimental observation so far and no related mechanism has been proposed to date.

In the present work, we prepared six metastable (Ti_0.615_Zr_0.385_)_100-3.9*x*_(Cu_2.3_Fe_1.6_)_*x*_ (0 ≤ *x* ≤ 1.5) alloys and found reversible partial SSA in certain compositions on cooling. The amorphous phase has a characteristic lenticular shape uniformly distributed inside metastable β-Ti grains along defined crystallographic directions. The amorphous phase apparently forms through a martensitic transformation in the crystalline solid as a result of local lattice shear. Simultaneously, the present SSA fulfils the criteria of congruent inverse melting. The fact that it proceeds catastrophically via a martensitic transformation sheds light onto fundamental phenomena of martensitic transformations, solid-state amorphization (SSA) and catastrophic melting.

## Results

### Morphology of the intragranular amorphous phase

**Fig. 1:**
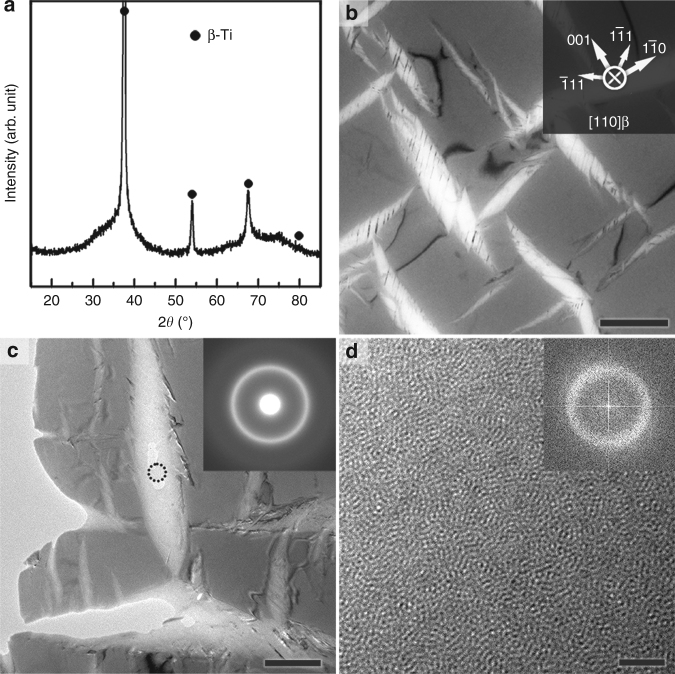
Microstructure of the as-cast Ti_59.1_Zr_37_Cu_2.3_Fe_1.6_ ingot. **a** Next to β-Ti reflections, a broad diffraction hump from an amorphous phase is found in the X-ray diffraction pattern. **b** Bright-field TEM micrograph revealing lenticular bright regions with their long axes preferentially oriented along <110>_β_ and <001>_β_. The lenses are embedded in a β-Ti matrix and are usually sheared multiple times along the <111>_β_ directions. **c** Bright-field TEM micrograph at a higher magnification and a SAED pattern (as inset) from the centre of the lenticular region (see circle). The halo corroborates the presence of an amorphous phase. **d** HRTEM image from the region marked in **c** with the corresponding FFT image as inset. Both clearly imply that the lenticular regions are mainly amorphous. The scale bars are 1 μm in **b**, 500 nm in **c**, and 2 nm in **d**

Even though the following results and discussion also hold for several other alloys, we only concentrate on Ti_59.1_Zr_37_Cu_2.3_Fe_1.6_ (*x* *=* 1) as a case study here. Figure 1a shows the X-ray diffraction (XRD) pattern of the Ti_59.1_Zr_37_Cu_2.3_Fe_1.6_ ingot. Next to the diffraction peaks of body-centred cubic (bcc) β-Ti, the broad scattering contribution of an amorphous phase is seen, which is consistent with the high-energy XRD of this sample (Supplementary Fig. 1). In the transmission electron microscope (TEM) micrograph of this sample (Fig. 1b), characteristic bright lenticular plates inside a β-Ti grain are visible. The long axis of these plates is oriented along the crystallographic <110>_β_ or <001>_β_ directions and several strips in <111>_β_ directions span the lenticular plates. At a higher magnification (Fig. 1c), the selected area electron diffraction (SAED) pattern from the centre of the lenticular plates produces a halo (inset in Fig. 1c). In combination with the characteristic maze-like pattern in the high-resolution TEM (HRTEM) image (Fig. 1d), this confirms the presence of an amorphous phase. As-cast Ti_59.1_Zr_37_Cu_2.3_Fe_1.6_ rods with a diameter of 3 mm or 8 mm show similar microstructures (Supplementary Fig. 2) and, consequently, the cooling rates used in this work (1.6 to 440 K s^−1^, see Methods) do not obviously affect the formation of the amorphous phase. To eliminate the possibility of preparation artefacts, the TEM specimens were additionally thinned by gentle ion-milling and the observed microstructures (Supplementary Figs. 3 and 4) are identical. The thickness of the amorphous phase and the adjacent β-Ti matrix as measured by electron energy loss spectroscopy (EELS) is 154 and 223 nm, respectively (Supplementary Fig. 4). Hence, one can exclude that the sample preparation for transmission electron microscopy selectively amorphizes α′ or α″ martensite. This conclusion is corroborated by the fact that in the case of Ti_60.3_Zr_37.7_Cu_1.2_Fe_0.8_ (*x* = 0.5) and Ti_59.6_Zr_37.3_Cu_1.8_Fe_1.3_ (*x* = 0.8) both crystalline martensites can be revealed in the TEM micrograph (see Supplementary Fig. 7).

**Fig. 2:**
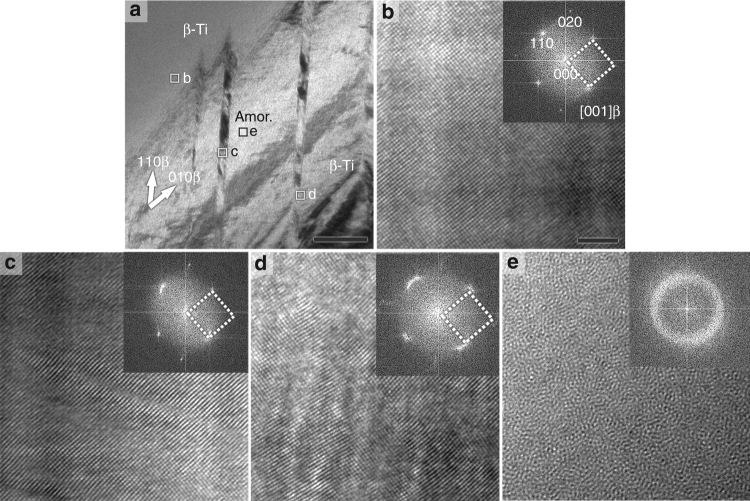
Detailed microstructure of an as-cast Ti_59.1_Zr_37_Cu_2.3_Fe_1.6_ rod with a diameter of 8 mm. **a** The bright-field TEM micrograph exhibits a representative lenticular region containing shear steps inside the β-Ti grain. The strips are parallel to <111>_β_, which corresponds to <110>_β_ when the crystal is viewed along [001]_β_. **b**–**e** HRTEM images taken from those regions marked b–e in **a**, respectively, with insets of the corresponding FFT images. **b** The matrix surrounding the lenses consists of unstrained β-Ti. **c**, **d** The FFT spots split, indicating a highly distorted β phase. **e** The maze-like pattern and the halo in the FFT image imply the presence of an amorphous phase. The scale bar in **a** represents 100 nm, and the scale bar in **b** 2 nm, which also applies to **c**–**e**

Figure 2a shows a section of a lenticular region in a Ti_59.1_Zr_37_Cu_2.3_Fe_1.6_ rod with a diameter of 8 mm. The strips are parallel to <110>_β_ under a viewing direction of [001]_β_ and the shear steps they accompany are evident. HRTEM images (Fig. 2b–e) were recorded from the regions marked in Fig. 2a and the corresponding fast fourier transform (FFT) images (insets) were generated. It is clear that the β-Ti matrix has an undistorted bcc lattice (Fig. 2b), while the β-Ti diffraction spots from the strip are broadened and split (Fig. 2c) indicating significant lattice distortion. An even more severe lattice distortion is found in the interface region between the strip and the β-Ti matrix (Fig. 2d). In the brighter region near the strips (Fig. 2e), a maze-like pattern is revealed and the FFT image only shows a halo, proving the presence of an amorphous phase.

The compositions of the amorphous phase and of the β-Ti matrix in the Ti_59.1_Zr_37_Cu_2.3_Fe_1.6_ rod with a diameter of 8 mm were measured by energy-dispersive X-ray spectroscopy (EDS) in the TEM and the average compositions are listed in Table 1. Both the amorphous phase and the β-Ti matrix have identical compositions, so that the current β-Ti grains apparently melt congruently from within.

**Table 1:** Mean composition of β-Ti and the amorphous regions in the ingot of Ti_59.1_Zr_37_Cu_2.3_Fe_1.6_ measured by EDS in the TEM

Region	Composition (at.%)			
	Ti (±0.8)	Zr (±0.8)	Cu (±0.2)	Fe (±0.2)
β-Ti	59.4	36.8	2.2	1.6
Amorphous	58.5	37.9	2.1	1.5

The β-Ti grains partially “melt” congruently from within

### Phase transformations and reversibility of amorphization

**Fig. 3:**
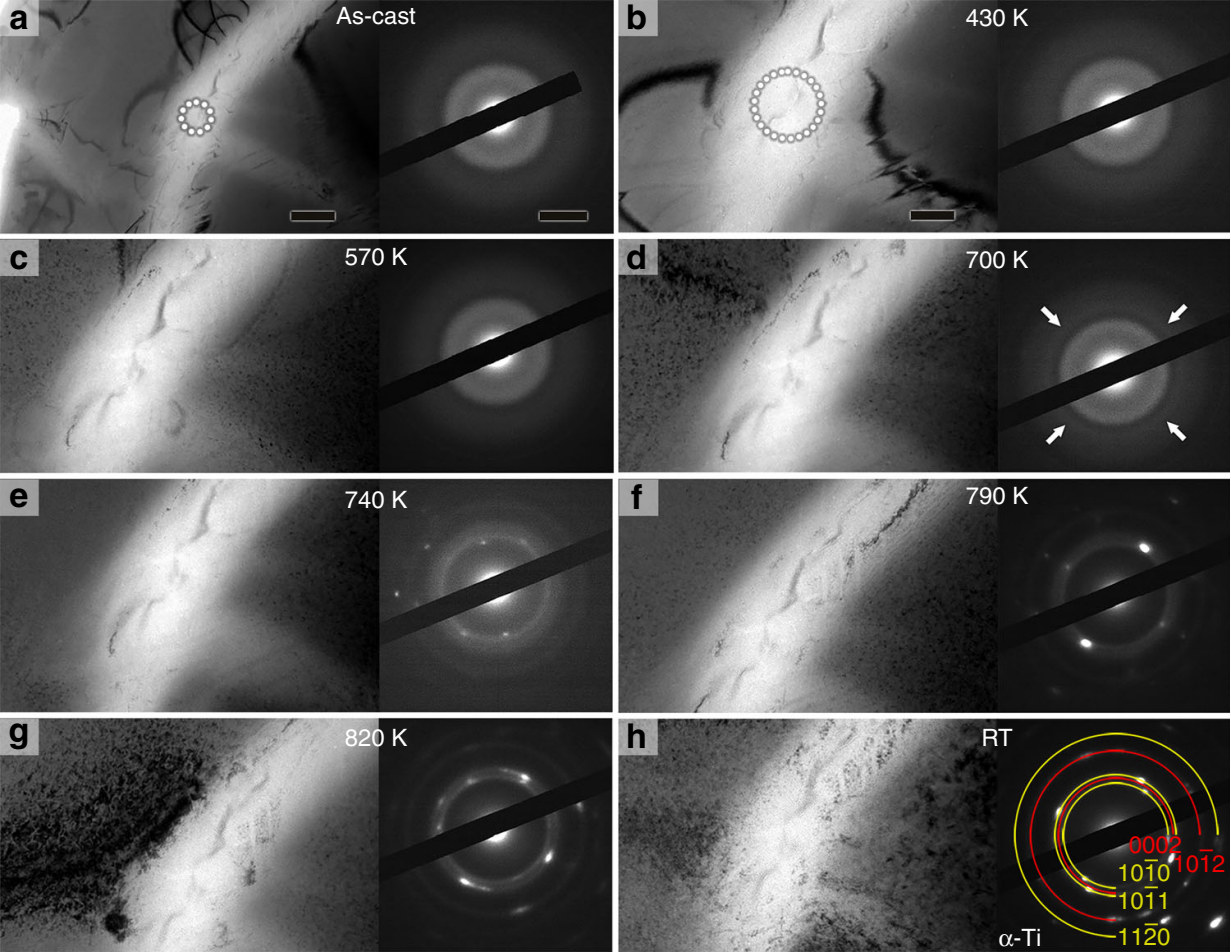
Microstructure and SAED patterns of Ti_59.1_Zr_37_Cu_2.3_Fe_1.6_ rod with a diameter of 8 mm during in situ heating in the TEM. **a** As-cast state. **b**–**g** In situ heating to temperatures below the α→β transition temperature. The amorphous phase begins to transform to α-Ti at around *T* = 700 K. **h** After cooling to room temperature (RT), α-Ti, the equilibrium phase, is only observed. Scale bars in **a** represent 200 nm for the micrograph and 2 nm^−1^ for the SAED pattern, respectively. Scale bar in **b** is 100 nm which also applies to **c**–**h**

In the following we present the crystallization behavior of the intragranular amorphous phase during in situ heating in the TEM. Prior to heating, the SAED pattern taken from the marked bright lenticular region (dotted circle in Fig. 3a) shows that it is fully amorphous. And no obvious microstructural change can be observed during heating to *T* = 430 K (Fig. 3b). At *T* = 570 K, very small dark spots appear in the β-Ti matrix, as shown in Fig. 3c. These small spots are isothermal ω particles. Still, the amorphous region does not undergo any obvious structural changes until the temperature reaches *T* = 700 K (Fig. 3d) and a sharp diffraction ring emerges in the SAED pattern in Fig. 3d (see arrows). At *T* = 740 K, the diffraction pattern is mainly composed of multiple rings from polycrystalline phases (Fig. 3e). Continuous heating up to *T* *=* 820 K (below the α → β transition temperature) increases the diffraction intensities of multiple rings but leaves their positions unaltered. The microstructure as well as the diffraction pattern does not undergo any additional transformations on cooling to room temperature (Fig. 3f–h). The prevailing crystalline phase is identified as α-Ti, which, hence, represents the equilibrium phase in the current alloy at temperatures below the α→β transition temperature.

**Fig. 4:**
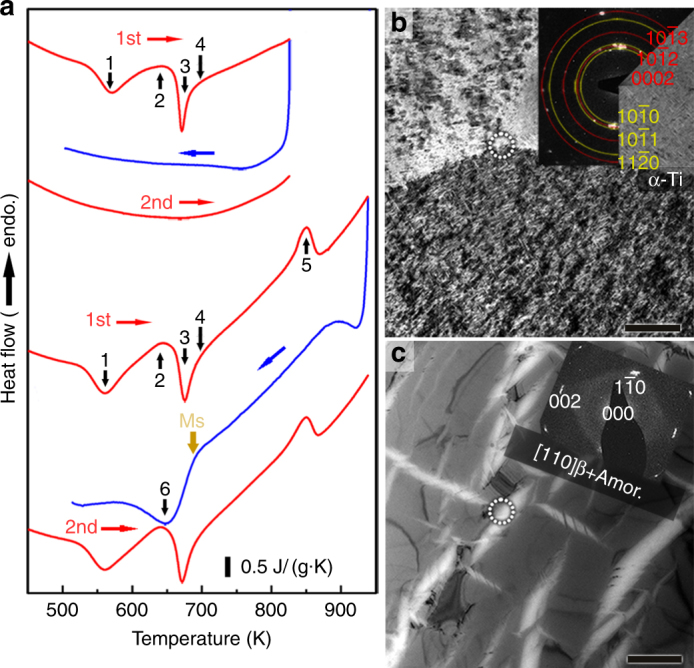
Cyclic differential scanning calorimetry of a Ti_59.1_Zr_37_Cu_2.3_Fe_1.6_ rod with a diameter of 8 mm and microstructures after different anneals. **a** When the as-cast alloy is heated to 823 K, four phase transformations proceed (1: β→β + ω, 2: ω→β, 3: β→α, 4: glass→α). No transformations occur on cooling or immediate reheating. During heating the as-cast alloy to 943 K, the β-transus temperature (5: α→β) is crossed. Subsequent cooling results in an exothermic event below 695 K. Since the same phase transformations take place on reheating like in the as-cast state, it reveals that this exothermic reaction (6) originates from partial amorphization of β-Ti (β→β + amorphous). **b** The bright-field TEM micrograph of the DSC sample annealed at 823 K and the SAED pattern from the marked region (inset) proves the presence of stable α-Ti. **c** After annealing at 943 K, the microstructure and the SAED pattern (inset) are identical to the as-cast state, which show β-Ti and the amorphous phase. Scale bars in **b**,** c** correspond to 1 μm

Differential scanning calorimetry (DSC) traces of a Ti_59.1_Zr_37_Cu_2.3_Fe_1.6_ rod with a diameter of 8 mm are shown in Fig. 4a. When the as-cast sample is heated to *T* = 823 K, which is below the α→β transition temperature (β-transus = 875 K), several thermal events are discernible, which were additionally examined ex situ by XRD (Supplementary Fig. 5). The sequence of phase transformations on heating is β→β+ω→β→α and above 725 K: amorphous→α (see Fig. 3). No transformation can be detected on cooling or reheating (Fig. 4a). The TEM micrograph of Ti_59.1_Zr_37_Cu_2.3_Fe_1.6_ after the thermal cycle mainly discloses α-Ti, as shown in Fig. 4b. In the case Ti_59.1_Zr_37_Cu_2.3_Fe_1.6_ is heated to *T* = 943 K (above the β-transus), however, an exothermic event is detected at 695 K on subsequent cooling. This anneal restores all transformations found in the as-cast state during subsequent heating (Fig. 4a). Additionally, the as-cast (Fig. 1b) and the thermally cycled microstructure (Fig. 4c) are two of a kind, which substantiates the reversibility of amorphization in the current alloy. Besides these obvious microstructural indicators, one can rule out that the exothermic event on cooling is the signature of the β-to-ω transformation for another reason. The endothermic ω-to-β transformation has its onset temperature just above 600 K (Supplementary Fig. 5). As for any martensitic transformation, the β-to-ω transformation on cooling must occur at lower temperatures than the reverse reaction on heating. The exothermic reaction starting at 695 K during cooling consequently indicates inverse melting of metastable β-Ti.

## Discussion

**Fig. 5:**
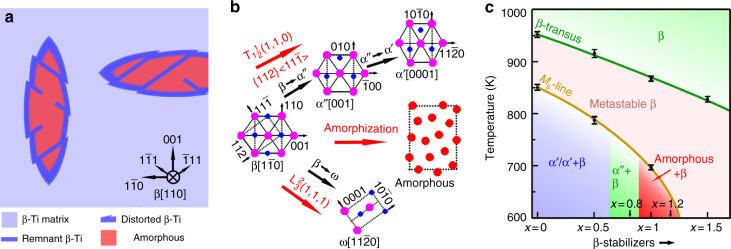
Schematic depiction of the martensitic amorphization. **a** Sketch of the observed microstructure illustrating the lenticular amorphous regions and their preferred orientations along <110>_β_ or <001>_β_. A ${\mathrm{\{ 112\} }}\left\langle {{\mathrm{11}}\overline {\mathrm{1}} } \right\rangle$ lattice shear results in remnant β-Ti strips parallel to $\left\langle {{\mathrm{11}}\overline {\mathrm{1}} } \right\rangle$ and distinct shear steps in the lenticular regions. **b** Crystallography of conventional martensitic transformations from β (${\mathrm{Im}}\overline {\mathrm{3}} {\mathrm{m}}$) to α′ (${\mathrm{P6}}_{\mathrm{3}}{\mathrm{/mmc}}$), α″ (Cmcm), and ω (${\mathrm{P6/mmm}}$). By an irregular atomic displacement, the β lattice can collapse into an amorphous phase. **c** Metastable phase diagram with DSC-measured data of (Ti_0.615_Zr_0.385_)_100-3.9*x*_(Cu_2.3_Fe_1.6_)_*x*_ (0 ≤ *x* ≤ 1.5) alloys during cooling. The error bars indicate the standard deviation of the DSC data during heating (β-transus) and cooling (*M*_s_-line)

The characteristics of the present polymorphic SSA strongly differ from previous reports^4,22,43^. Here, the glassy phase is confined in lenticular plates, which are distributed uniformly inside the grains rather than at grain boundaries (Supplementary Fig. 6). Additionally, the principal axes of the lenticular plates have a well-defined orientation with respect to the surrounding β-Ti crystal, as depicted in Fig. 5a. There is no obvious difference in the amorphous volume fraction in samples cooled at different rates. Moreover, the lenticular amorphous phase bears a striking resemblance to conventional α′-Ti or α″-Ti martensite (Supplementary Fig. 7b, c). This ultimately suggests that the current polymorphic amorphization is neither initiated by nucleation at defects nor governed by diffusion. Instead, the transformation is of martensitic nature, which inflicts the term “amorphous martensite.”

In the following, we propose a mechanism for the amorphization inside the metastable β-Ti grains. Generally, metastable β-Ti can transform in a displacive manner either to the α′/α″ martensite or the ω phase^36^. The crystallography of these transformations is sketched in Fig. 5b. If metastable β-Ti is lean in β-stabilizing elements, a phonon anomaly, ${\mathrm{T}}_1\frac{1}{2}{\mathrm{(1,1,0)}}$, combined with a $\left\{ {{\mathrm{112}}} \right\}\left\langle {{\mathrm{11}}\overline {\mathrm{1}} } \right\rangle$ lattice shear and an atomic shuffle transforms the bcc lattice to hexagonal α′ martensite^40^. If β-Ti contains a larger amount of β-stabilizing elements, the lattice distortion and shuffle toward α′ martensite are incomplete, and the orthorhombic α″ martensite forms instead^40,44^. Another phonon anomaly, ${\mathrm{L}}\frac{{\mathrm{2}}}{{\mathrm{3}}}{\mathrm{(1,1,1)}}$, transforms the metastable β-Ti to the ω phase^40^. Only for alloy compositions with an appropriate content of β-stabilizers can martensitic amorphization occur, due to relatively large local lattice strains, which impede the ideal cooperative movement of atoms required to form the α′/α″ martensite or the ω phase. Instead, the atoms are displaced non-uniformly and the outcome is a structure deprived of any long-range order (Fig. 5b). The lenticular plates are preferably aligned along the <110>_β_ and <001>_β_ directions, which reflects the urge of the crystal lattice to minimize the local strain energy^45^ during the martensitic transformation. The ${\mathrm{\{ 112\} }}\left\langle {{\mathrm{11}}\overline {\mathrm{1}} } \right\rangle$ lattice shear^40,44^ is then responsible for the observed remnant β-Ti strips along the <111>_β_ directions: During cooling of the Ti_59.1_Zr_37_Cu_2.3_Fe_1.6_ alloy, the β-Ti lattice becomes instable below the β-transus temperature and the lattice begins to shear locally on certain {112} planes along the $\left\langle {{\mathrm{11}}\overline {\mathrm{1}} } \right\rangle$ directions. Adjacent {112} planes also begin to shear on the continued decrease of temperature. The local strain on the initial shear planes is relatively small but it rises with increasing distance between the active planes and the initial planes. Once the local strain reaches a critical value, the sheared β-Ti planes collapse into the disordered amorphous phase. The initial, less sheared planes remain during the martensitic amorphization and eventually connect two shear steps (the positions where the planes begin to shear) on both sides of the lenticular amorphous regions. A combination of elastic softening and lattice shear, hence, accounts for the formation of martensite or an amorphous phase alike. This unexpected inverse melting phenomenon suggests that the shear instability of the crystal lattice must also define the maximum thermal stability of crystalline solids on superheating.

The formation of an amorphous martensite is restricted to a relatively narrow compositional regime, as shown in Fig. 5c and Supplementary Fig. 7, and an explanation for this shall be given here. With an increasing amount of β-stabilizing elements, the *M*_s_-line plunges steeply^44^ and martensite cannot be obtained at room temperature, e.g., in Ti_57.9_Zr_36.3_Cu_3.4_Fe_2.4_ (*x* = 1.5) (Supplementary Fig. 7). Only at intermediate contents of β-stabilizers can the amorphous phase compete with the formation of α′, α″, or ω. Thereby, its free energy has to be very close to that of the crystalline phases. This condition is only satisfied in Ti_59.1_Zr_37_Cu_2.3_Fe_1.6_ (*x* = 1) and Ti_58.6_Zr_36.7_Cu_2.7_Fe_1.9_ (*x* = 1.2). The contribution of the elastic strain energy to the free energy locally renders the amorphous phase thermodynamically more favorable than α′, α″, or ω. When the content of β-stabilizing elements is relatively low, crystalline martensite is stabilized with respect to the amorphous phase and either α′ or a mixture of β+α′/α″ forms (Supplementary Fig. 7), as summarized in the schematic metastable phase diagram in Fig. 5c. Because the configurational entropy of an amorphouse/liqud phase is higher than that of its crystalline counterpart, the spontaneous congruent melting of a crystal into an amorphous phase during cooling seems paradoxical from a thermodynamic point of view (Kauzmann paradox)^46^. It is likely that pronounced chemical ordering^25,47,48^ in the current amorphous phase resolves this apparent violation of thermodynamic laws and renders amorphization possible. Therefore, we speculate that martensitic amorphization can only occur in sufficiently complex alloys. Another prerequisite is the relatively open crystal lattice as in a bcc structure. Its vibrational entropy is larger compared to close-packed structures and favors inverse melting^25,48^. Otherwise, the decrease in entropy by chemical ordering would be insufficient to overcome the increase in configurational entropy during amorphization^25^.

The current martensitic amorphization appears to be a universal phenomenon and it has been detected in as-cast Ti_59.1_Zr_37_Cu_2.3_Co_1.6_ and Ti_59.1_Zr_37_Cu_2.3_Ni_1.6_ alloys (Supplementary Fig. 8). The fact that polymorphic solid-state amorphization (i.e., congruent inverse melting) can proceed via a martensitic transformation supports Born’s criterion for melting^30^, i.e., that elastic softening and lattice shear account for catastrophic melting inside a crystal. The present experiments not only propose an alternative solid-state amorphization mechanism, but also reveal the fundamental connection between the mechanisms underlying martensitic transformations and catastrophic melting.

## Methods

### Alloy preparation

The compositions of the alloys investigated in the current work include (Ti_0.615_Zr_0.385_)_100-3.9*x*_(Cu_2.3_Fe_1.6_)_*x*_ (*x* = 0, 0.5, 0.8, 1, 1.2, and 1.5), Ti_59.1_Zr_37_Cu_2.3_Co_1.6_ and Ti_59.1_Zr_37_Cu_2.3_Ni_1.6_. All compositions are given in atomic percent. Alloy ingots of these alloys with a mass of about 100 g were prepared by arc melting the respective elements (purities higher than 99.9 wt.%) in a Ti-gettered high-purity argon atmosphere (Hotstar arc melter, China). The ingots were remelted three times in order to assure chemical homogeneity. In addition, pieces of the ingots were arc-melted (Edmund Bühler, Germany) and quenched in water-cooled copper moulds to produce rods with a diameter of 3 or 8 mm.

### Estimation of cooling rates

According to previous experiments^49^, the cooling rate of a typical 100 g ingot within the used arc melter (Hotstar) is roughly equivalent to that of an as-cast rod with a diameter of 50 mm. As proposed by Lin and Johnson^50^, the cooling rates as a function of the sample (rod) radius, *R*, can be calculated as:

1 $$\dot T = \frac{{{\rm d}T}}{{{\rm d}t}} \approx \frac{{\Delta T}}{\tau } = \frac{{K\Delta {\it{T}}}}{{CR^{\mathrm{2}}}}$$

where *K* is the thermal conductivity, *C* the heat capacity per unit volume and Δ*T* is the temperature interval between the melting temperature and the glass-transition temperature. For Ti/Zr-based alloys, Δ*T* ≈ 400 K, *K* ≈0.1 W cm^−1^ K^−1^, and *C* ≈ 4 J cm^−3^ K^−1^, therefore:

2 $$\dot T{\mathrm{(K s}}^{{\mathrm{ - 1}}}{\mathrm{)}} \approx 10R^{{\mathrm{ - }}2}{\mathrm{(cm)}}.$$

From equation (2), the cooling rates of as-cast rods with a diameter of 3 or 8 mm, and the 100 g ingots are 440, 60, and 1.6 K s^−1^, respectively.

### TEM sample preparation

Cross-sectional slices with a thickness of ~100 μm were cut from the rods and ingots. These slices were ground to a final thickness of about 50 μm and small disks with a diameter of 3 mm were punched out. In the current study, both twin-jet electrolytic thinning and ion-milling were employed to prepare the TEM specimens. The results shown in the publication were all obtained from electrolytically thinned specimens. Twin-jet electrolytic thinning of the TEM specimens was performed in a Struers TenuPol-5 using a solution of 30 ml perchloric acid +175 ml 1-butanol +295 ml methanol at 248 K and a constant voltage of 20 V. After perforation, the TEM specimens were cleaned with ethanol. For ion-milling, the disks were dimpled using a Gatan dimpler grinder (Model 656) until the thickness of the centre reached a value of about 10 μm. The dimpled disks were then ion-milled in a Gatan 691 or Gatan 695 (PIPS II) precision ion polishing system with liquid nitrogen cooling. The disks were first ion-milled at 3.5 kV, 10 mA and an angle of 8 ° up to perforation, then the disks were ion-milled at 3.0 kV, 10 mA and an angle of 4 ° for 20 min. Additionally, the TEM samples were treated in a Fischione plasma cleaner (model 1020) for 10 min to remove possible amorphous layers at the surface, which might form during ion-milling. All TEM specimens were stored in high vacuum prior to TEM observation.

### TEM characterization

The TEM observations were carried out in a JEM-2100 (JEOL) transmission microscope and a Tecnai F30 (FEI) transmission microscope. The JEM-2100 was operated at 200 keV and the smallest diameter of the apertures (120 nm) was used for obtaining the SAED patterns. The Tecnai F30 was operated at 300 keV and the aperture diameter used for the SAED patterns was 200 nm. Moreover, the Tecnai F30 was equipped with a scanning transmission electron microscopy (STEM) system, an EDS unit (EDS, Oxford) and a high-angle annular dark field detector. The HRTEM images were obtained using the Tecnai F30, and the corresponding FFT images were calculated with the help of the Gatan DigitalMicrograph software. The in situ heating TEM characterization of the as-cast Ti_59.1_Zr_37_Cu_2.3_Fe_1.6_ rod with a diameter of 8 mm was carried out in the JEM-2100 microscope. The specimens were heated to different temperatures at a heating rate of 10 K min^−1^, and the temperature was then held constant for 2 min in order to record the TEM micrographs and the corresponding SAED patterns. The thickness of the TEM specimens was determined in a FEI Titan G2 300 equipped with an EELS unit.

### X-ray diffraction (XRD)

The slices for XRD were cut from the centre of the Ti_59.1_Zr_37_Cu_2.3_Fe_1.6_ ingot where the cooling rate is expected to be the lowest and cut transversely from all (Ti_0.615_Zr_0.385_)_100-3.9*x*_(Cu_2.3_Fe_1.6_)_*x*_ rods with a diameter of 8 mm. The surfaces of the slices were subsequently ground and polished. The XRD measurements were performed using a Phillips PW1050 with Cu-Kα radiation (*λ* = 0.15406 nm). The high-energy XRD data of the Ti_59.1_Zr_37_Cu_2.3_Fe_1.6_ ingot was collected at the beamline 11-ID-C at the Advanced Photon Source, Argonne National Laboratory, USA.

### Differential scanning calorimetry (DSC)

Slices with a weight of about 35 mg were cut from the Ti_59.1_Zr_37_Cu_2.3_Fe_1.6_ rod with a diameter of 8 mm for DSC characterization. The DSC measurements were performed using a Perkin-Elmer DSC7 at a heating rate of 20 K min^−1^ and a cooling rate of 100 K min^−1^. In the case the target temperatures were lower than 870 K, the samples were heated in aluminium crucibles. Otherwise, the samples were heated in alumina crucibles. For the cyclic DSC experiments, the samples were heated and cooled a second time using identical parameters as in the first run. Moreover, samples were annealed in the DSC in order to investigate the phase evolution by XRD. For this, the samples were heated to their final temperature and then immediately cooled with the maximum cooling rate possible of 100 K min^−1^.

### Data availability

The data that support the findings of this study are available from the corresponding author on request.

## Electronic supplementary material

Supplementary Information
