# Supplementary material for: Amorphous martensite in β-Ti alloys
Source: Nat Commun. 2018 Feb 6;9:506. doi: 10.1038/s41467-018-02961-2 (PMC5802800; doi:10.1038/s41467-018-02961-2)
Supplement: Supplementary file 1 — Supplementary Information [file 41467_2018_2961_MOESM1_ESM.pdf]

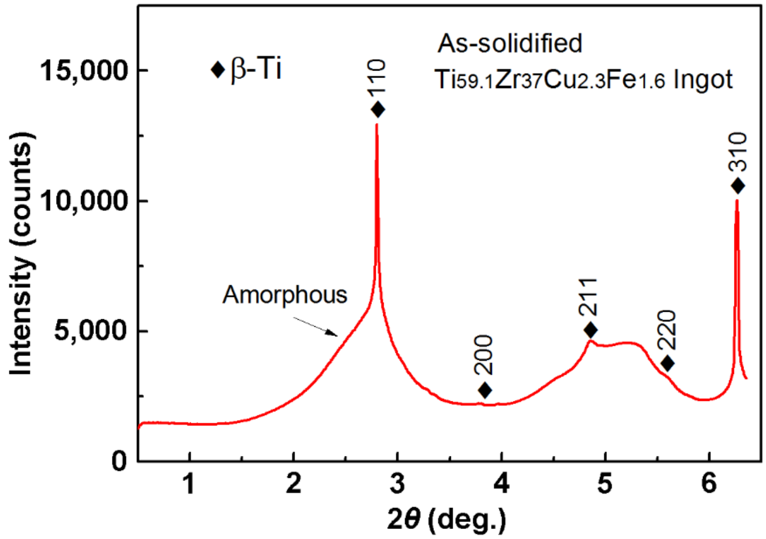

Supplementary Figure 1 | High-energy X-ray diffraction pattern of the $Ti_{59.1}Zr_{37}Cu_{2.3}Fe_{1.6}$ ingot. Two diffuse maxima at around 2θ=2.5º and around 2θ=5.2º are visible, which indicate the presence of an amorphous phase.

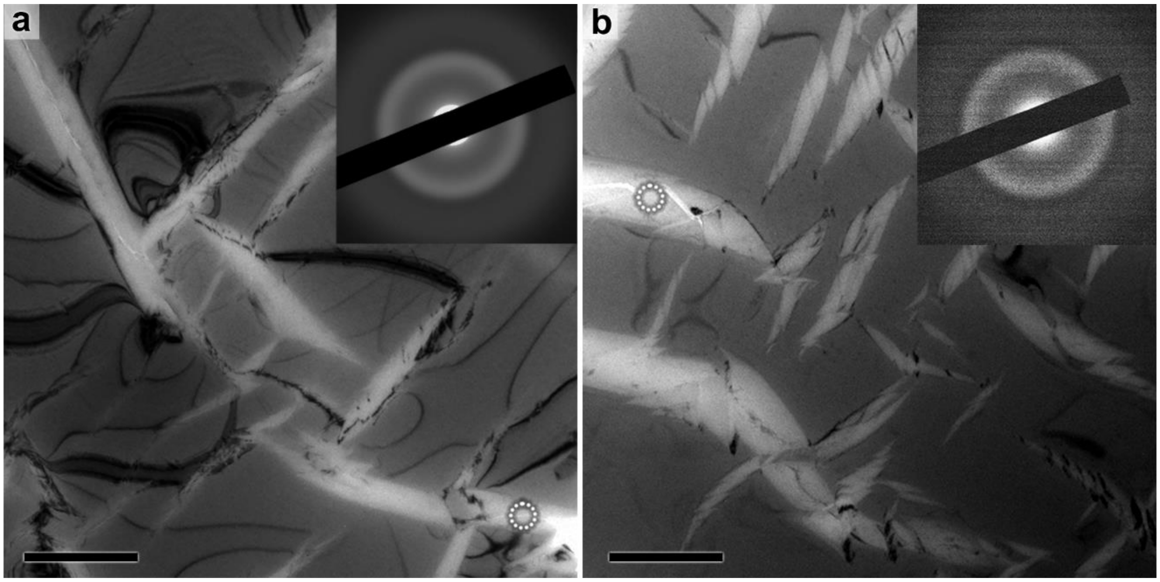

Supplementary Figure 2 | Bright-field TEM micrographs of as-cast $Ti_{59.1}Zr_{37}Cu_{2.3}Fe_{1.6}$ rods with different diameters. (**a**) Rod with a diameter of 3 mm and (**b**) rod with a diameter of 8 mm. Both samples have a microstructure identical to the microstructure found in the ingot (Fig. 1b) consisting of bright lenticular regions embedded in a β-Ti matrix. The SAEDs (insets) were taken from the locations indicated by the circles and reveal the amorphous structure inside the lenticular regions. The size of the lenticular plates and their volume fraction are similar in these samples. The present cooling rates (between 1.6 K s^{-1} and 440 K s^{-1}) are sufficient to suppress the formation of α-Ti and intermetallic phases in equilibrium. Scale bars in both micrographs represent 1 μm.

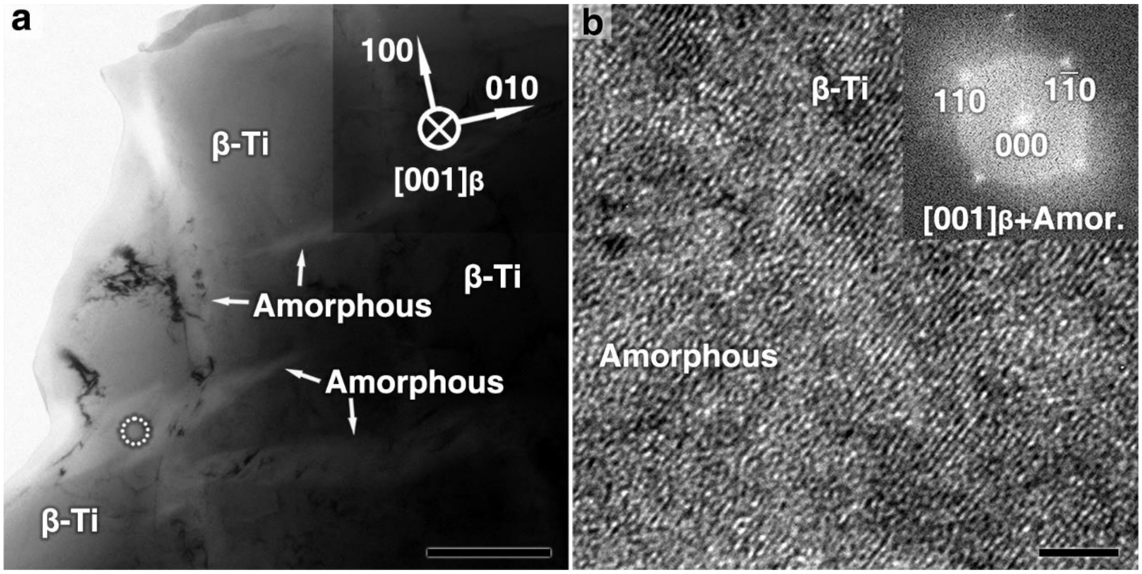

Supplementary Figure 3 | $Ti_{59.1}Zr_{37}Cu_{2.3}Fe_{1.6}$ specimens prepared by ion milling. (**a**) A typical bright-field TEM micrograph of a rod with a diameter of 8 mm. Lenticular bright regions containing several β-Ti strips are embedded in the β-Ti grain. (**b**) The HRTEM image was taken near the edge of a strip (marked by the circle in **a**) and the inset shows the according FFT image. The body-centred cubic lattice gradually collapses starting from the β-Ti strip (upper right in **b**) to the more uniform bright side (lower left in **b**). This confirms that the strips in the lenticular regions are distorted β-Ti and the uniform bright regions are amorphous. Compared to the electrolytically thinned TEM samples (Fig. 1b), the ion-milled samples produce an identical microstructure but with less pronounced contrast due to a similar thickness of the amorphous regions and the β-Ti matrix. Scale bars: 500 nm in **a** and 2 nm in **b**.

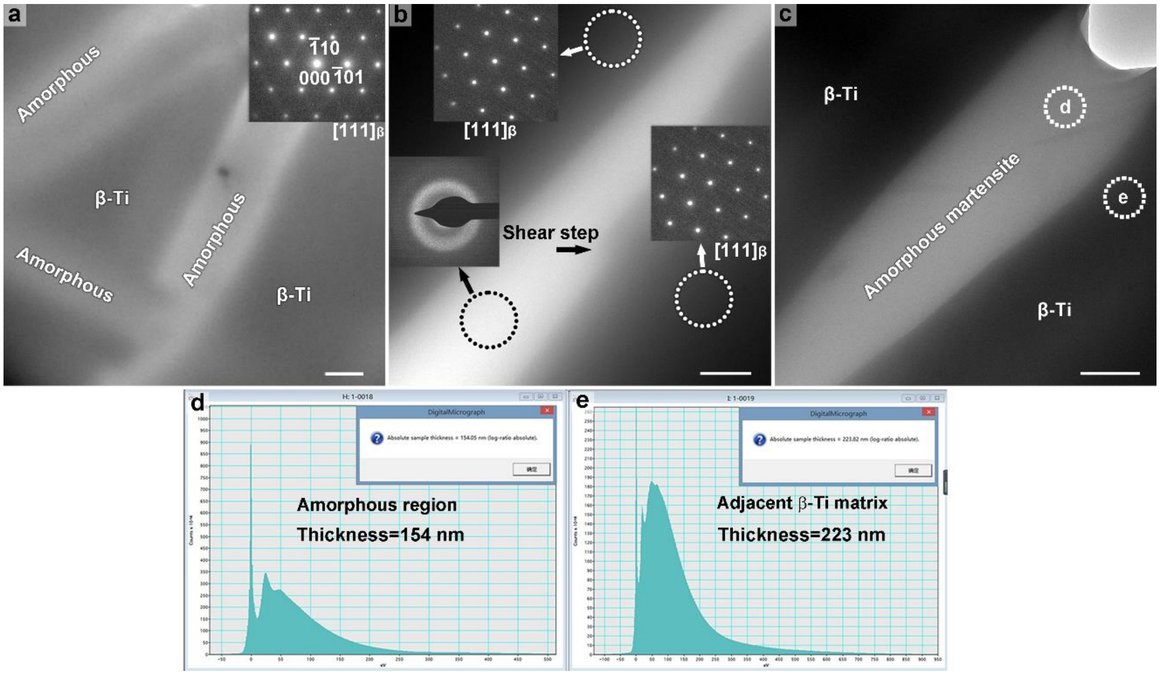

Supplementary Figure 4 | TEM characterization of an ion-milled specimen of the $Ti_{59.1}Zr_{37}Cu_{2.3}Fe_{1.6}$ ingot. (**a**) TEM micrograph showing amorphous laths embedded inside a β-Ti grain under a zone axis of $[111]_{\beta}$. (**b**) TEM micrograph with selected area electron diffraction patterns as insets, taken from the indicated regions. A shear step at the interface is clearly seen. (**c**) TEM micrograph of another region used for analysing the sample thickness by means of electron energy loss spectroscopy (EELS). (**d**) and (**e**), The EELS spectra obtained from the amorphous martensite and the β-Ti matrix in **c**. The calculated thickness of the amorphous region and the crystalline matrix is 154 nm and 223 nm, respectively. All scale bars in **a-c** are 200 nm.

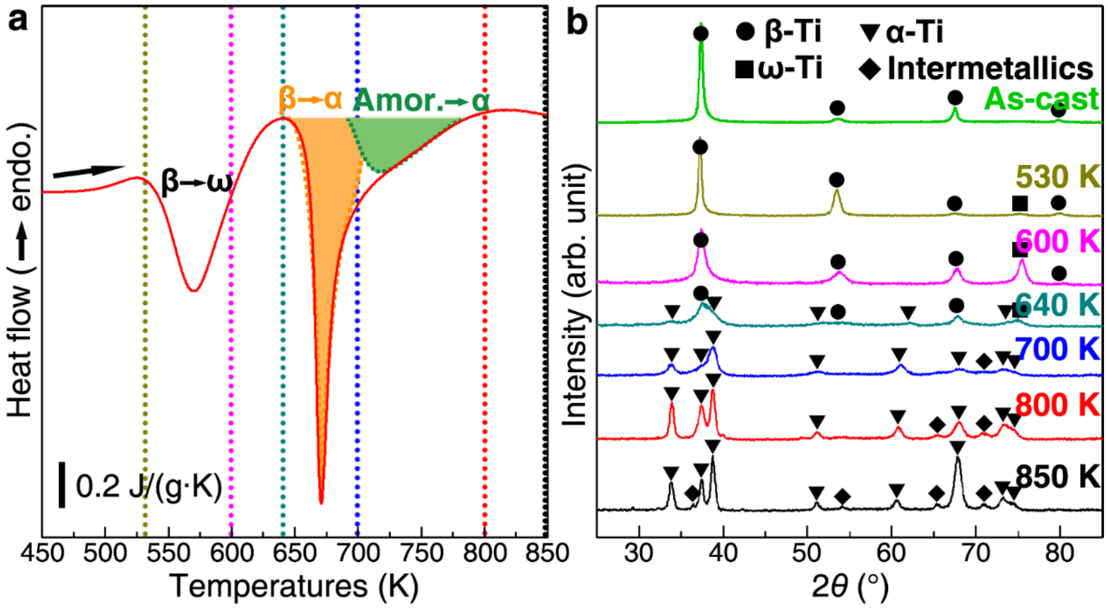

Supplementary Figure 5 | Phase transformations on heating in the as-cast $Ti_{59.1}Zr_{37}Cu_{2.3}Fe_{1.6}$ alloy. (**a**) DSC trace of the rod with a diameter of 8 mm recorded at a heating rate of 20 K min^{-1}. (**b**) X-ray diffraction (XRD) patterns of samples heated in the DSC to various temperatures indicated by the dotted lines in (a). At the onset of the first exothermic event (T = 530 K), the first weak reflections of the ω phase are detected. When the sample is heated to 600 K, the ω diffraction peak (at 2θ = 75 °) is relatively strong. In other words, the formation of the ω phase manifests itself in the first exothermic event having its peak at around 570 K. The ω phase becomes unstable as the temperatures rises and transforms back to the β phase, which causes the endothermic peak at T = 640 K. α-Ti reflections become visible at 640 K. At temperatures exceeding 700 K, only diffraction peaks of α-Ti and very weak reflections of the intermetallic phase $(Ti,Zr)_2(Cu,Fe)$ can be detected. The broad exothermic peak around 700 K in the DSC trace must thus be separated into two peaks: while the first represents the β→α transformation, the second peak at around 725 K is caused by the transformation of the amorphous phase to α-Ti.

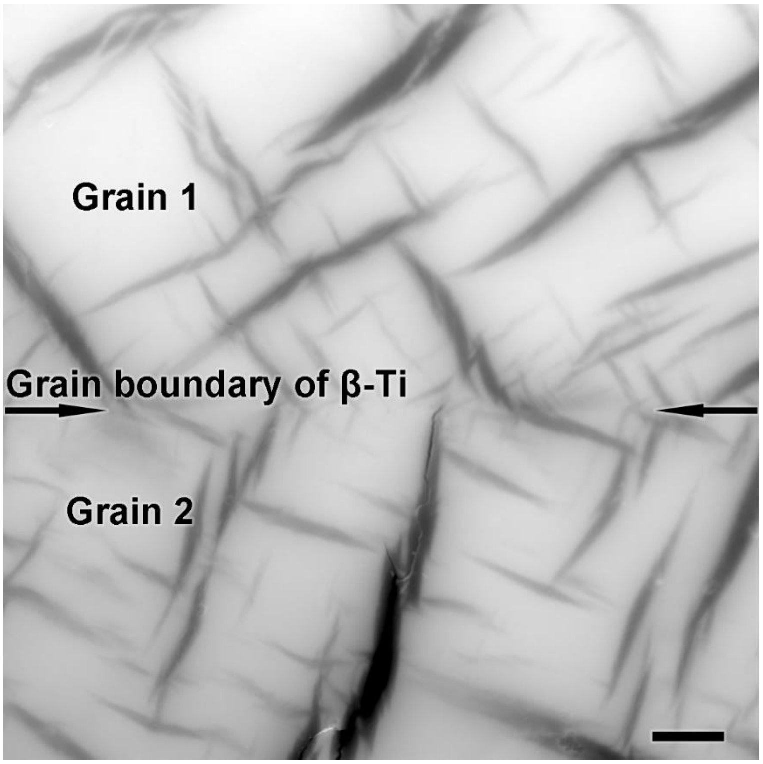

Supplementary Figure 6 | High-angle annular dark-field (HAADF) micrographs of a $Ti_{59.1}Zr_{37}Cu_{2.3}Fe_{1.6}$ rod with a diameter of 8 mm. Two β-Ti grains are shown. In either grain, lenticular amorphous regions exhibit the same orientation relationship with the surrounding β grain. Amorphous martensite locates inside β-Ti grains rather than at grain boundaries. Scale bar is 500 nm.

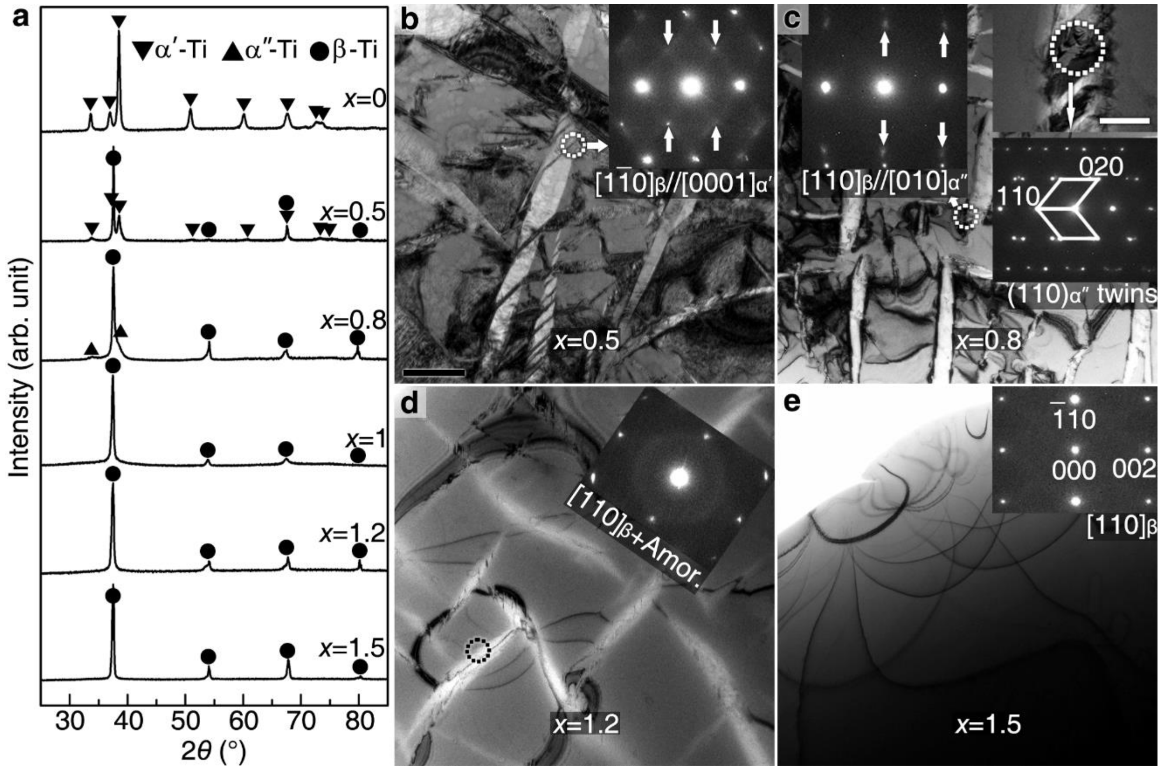

Supplementary Figure 7 | Phase formation in different as-cast $(Ti_{0.615}Zr_{0.385})_{100-3.9x}(Cu_{2.3}Fe_{1.6})_x$ rods with a diameter of 8 mm. (**a**) The X-ray diffraction patterns (XRD) at room temperature indicate that the crystalline phases change from α′→α′+β→α″+β→β with increasing Cu and Fe content. (**b**) The TEM micrograph of $Ti_{60.3}Zr_{37.7}Cu_{1.2}Fe_{0.8}$ (x = 0.5) also shows sheared lenticular plates but the material is fully crystalline and consists of α′-Ti, as indicated by the SAED pattern (see inset). (**c**) In the TEM micrograph of $Ti_{59.6}Zr_{37.3}Cu_{1.8}Fe_{1.3}$ (x = 0.8), the typical lenticular plates are more evident. In this alloy, they are composed of α″-Ti (see inset). (**d**) In $Ti_{58.6}Zr_{36.7}Cu_{2.8}Fe_{1.9}$ (x = 1.2) the presence of an amorphous phase in the lenticular plates is revealed by the corresponding SAED pattern (inset). (**e**) For $Ti_{57.9}Zr_{36.2}Cu_{3.5}Fe_{2.4}$ (x = 1.5), β-Ti is fully stabilized and is exclusively found. Scale bar in **b** is 1 μm, which also applies to **c**-**e**. The scale bar in upper inset of **c** is 200 nm.

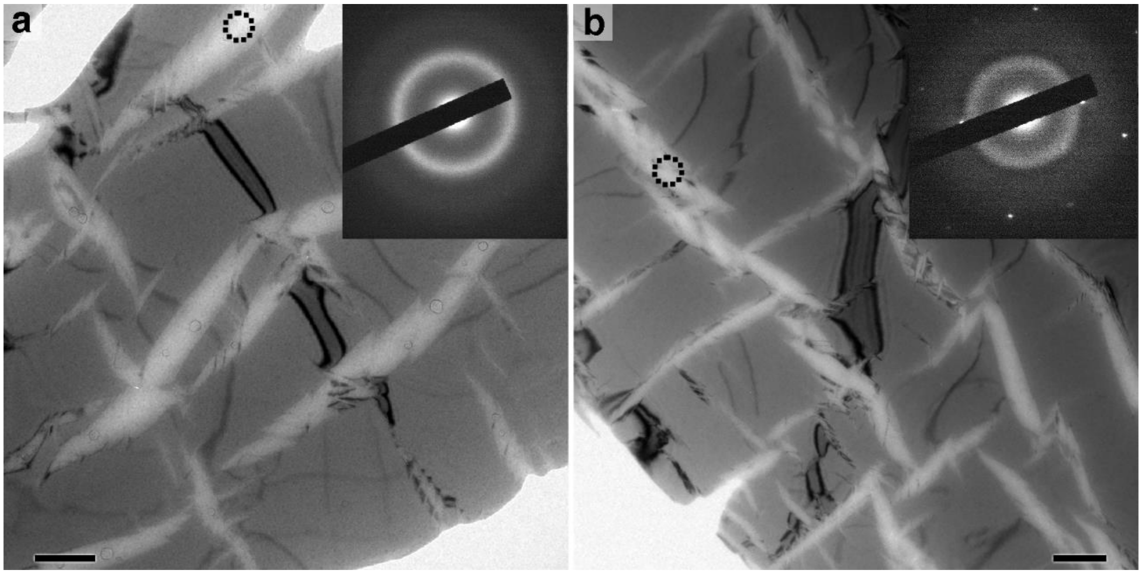

Supplementary Figure 8 | TEM micrographs of as-cast $Ti_{59.1}Zr_{37}Cu_{2.3}Co_{1.6}$ and $Ti_{59.1}Zr_{37}Cu_{2.3}Ni_{1.6}$ rods with diameters of 8 mm. (**a**) For $Ti_{59.1}Zr_{37}Cu_{2.3}Co_{1.6}$ as well as (**b**) $Ti_{59.1}Zr_{37}Cu_{2.3}Ni_{1.6}$, similar microstructures consisting of lenticular amorphous martensite embedded in β-Ti grains, are observed. The resemblance with the microstructure of $Ti_{59.1}Zr_{37}Cu_{2.3}Fe_{1.6}$ (Fig. 1b) is striking. Both scale bars represent 1 μm.
